# The Challenges of Living with Inflammatory Bowel Disease: Summary of a Summit on Patient and Healthcare Provider Perspectives

**DOI:** 10.1155/2016/9430942

**Published:** 2016-02-22

**Authors:** Judith Bray, Aida Fernandes, Geoffrey C. Nguyen, Anthony R. Otley, Joan Heatherington, Jennifer Stretton, Natasha Bollegala, Eric I. Benchimol

**Affiliations:** ^1^Judy Bray Consulting, 125 Grove Avenue, Ottawa, ON, Canada K1S 3A9; ^2^Crohn's and Colitis Canada, 600-60 Street Clair Avenue East, Toronto, ON, Canada M4T 1N5; ^3^Mount Sinai Centre for Inflammatory Bowel Disease, Mount Sinai Hospital, Department of Medicine, University of Toronto, 600 University Avenue, Toronto, ON, Canada M5G 1X5; ^4^Institute for Clinical Evaluative Sciences, G1 06, 2075 Bayview Avenue, Toronto, ON, Canada M4N 3M5; ^5^Division of Gastroenterology & Nutrition, IWK Health Centre, Department of Pediatrics, Dalhousie University, 5980 University Avenue, Halifax, NS, Canada B3K 6R8; ^6^University of Calgary Inflammatory Bowel Disease Clinic, Foothills Medical Centre, 1403-29 Street NW, Calgary, AB, Canada T2N 2T9; ^7^Division of Gastroenterology, St Joseph's Healthcare Hamilton, 50 Charlton Avenue East, Hamilton, ON, Canada L8N 4A6; ^8^Division of Gastroenterology, Women's College Hospital, 76 Grenville Street, Toronto, ON, Canada M5S 1B2; ^9^CHEO Inflammatory Bowel Disease Centre, Division of Gastroenterology, Hepatology and Nutrition, Children's Hospital of Eastern Ontario, Department of Pediatrics, School of Epidemiology, Public Health and Preventive Medicine, 401 Smyth Road, Ottawa, ON, Canada K1H 8L1

## Abstract

Canada has one of the highest rates of inflammatory bowel disease (IBD) and the disease represents a significant health, social, and economic burden. There is currently no cure for IBD, although earlier diagnosis and new therapies have improved the overall health outcomes and quality of life for patients. Crohn's and Colitis Canada is Canada's only national, volunteer-based charity dedicated to finding cures for IBD and improving the lives of those affected, through research, education, patient programs, advocacy, and increased awareness. On April 30, 2015, Crohn's and Colitis Canada hosted the “Patient and Healthcare Professional Summit on the Burden of Disease in IBD” to obtain a deeper understanding of the unmet needs of IBD patients and their caregivers. Through personal vignettes, patients articulated a pressing need to increase understanding of the challenges faced by people suffering from IBD among both health care professionals and the general public, develop best practices for navigating life transitions and addressing the unique challenges faced by children with IBD, and provide equitable access to appropriate, effective, and affordable treatments. The recommendations that emerged from the summit will inform about efforts to increase public awareness, inform about advocacy strategies, and contribute to the development of research priorities.

## 1. Background

Inflammatory bowel disease (IBD) represents an increasing health and economic burden. Canada has one of the highest rates of IBD, with more than 10,200 new cases diagnosed each year, an estimated 233,000 people living with IBD (including 5,900 children), and a projected cost to the Canadian economy of $2.8 billion per year [1]. The two most common forms of IBD, Crohn's disease (CD) and ulcerative colitis (UC), are lifelong diseases, characterized by periods of remission and flares, with symptoms that include bloody diarrhea, urgency, fatigue, weight loss, and abdominal pain [2]. Poorly controlled IBD can significantly impact a person's health and well-being, including their social, psychosocial, and economic status. Medical therapies produce sustainable long-term remissions in many patients, reducing the need for hospitalization and surgery and significantly improving the overall health and quality of life for those living with IBD [3, 4]. However, a person diagnosed with IBD is still likely to face challenges in successfully managing their disease, including inconsistencies in standards of care across the country, variability in support for patients and caregivers, and deficits in equitable, affordable access to drug therapies [1].

## 2. Patient and Healthcare Professional Summit on the Burden of Disease in IBD

On April 30, 2015, Crohn's and Colitis Canada convened a “Patient and Healthcare Professional Summit on the Burden of Disease in IBD.” The objective was to gather a small group of patients and healthcare professionals to share personal stories of the “real life” challenges of living with IBD. The goal was to obtain a deeper understanding of the unmet needs of IBD patients and their caregivers in order to increase public awareness, develop advocacy strategies, and identify research priorities. The day began with introductory presentations covering topics such as the incidence and epidemiology of IBD, access to care for IBD in Canada, treatment advances, the value of patient reported outcomes in monitoring disease activity and guiding clinical management, Crohn's and Colitis Canada IBD Survey [5], and key IBD research activities. The main focus of the day, however, was on gathering recommendations on how to improve the quality of life for people living with IBD.

## 3. Increasing Awareness: Understanding IBD

The impact of IBD on physical and mental health, social well-being, and economic stability of patients and their families is highly variable. Some patients reported excellent responses to initial treatments, followed by extended periods of remission, while others struggled to find effective therapies to control their disease. However, many IBD patients do not outwardly appear to be “sick” and so are sometimes treated with an insensitivity by the general public and even healthcare providers born of a fundamental lack of understanding of the disease. Many patients feel uncomfortable sharing their concerns and anxieties, preferring to keep their illness to themselves. This shroud of secrecy increases the burden on the patient and furthers the misconception that IBD is somehow under the patient's control and can be treated by reducing stress, changing diet, and adjusting lifestyle factors.

Lack of public education is a key factor in some of the daily challenges experienced by patients. Examples of such challenges included a university student who had to repeat an entire semester because of the urgent need to take an unexpected bathroom break during an exam, an adult IBD patient with active disease who had to beg to be allowed to use the bathroom in the business class section of a flight rather than wait in a long lineup, a patient who encountered questioning and resistance at airport security while trying to explain his need for baby cream and sanitary napkins, and several accounts of workplace discrimination following absences due to active disease.

Ironically, the IBD patient experience in healthcare facilities can be equally daunting. Some patients reported excellent care and support, even in small rural hospitals, whereas others reported long wait times for tests and specialist appointments compounded by a shortage of trained personnel, including specialized IBD nurses. Patients related stories of difficulties encountered with washroom access in the hospital setting, most of which were a result of nonspecialized nursing staff. For example, IBD patients admitted to a mixed hospital ward were often located far from a bathroom in order to give priority access to elderly patients or those with mobility issues. Patients explained that nurses do not always appreciate that IBD patients may need bathroom access 15–20 times a day and may not be able to walk long distances or stand in line for a toilet, even if they are young and mobile. For pediatric patients who may be too embarrassed to speak up for themselves, it is especially critical that nursing staff understand the social consequences for a patient with active disease requiring immediate bathroom access.

*Recommendations*. Patients called for more hospitals to have counsellors, social workers, and mental health professionals trained to understand the stress experienced by IBD patients and the concept of uncertainty that causes anxiety. These professionals offer a valuable support to patients and can help develop individualized strategies to reduce anxiety, depression, and other mental health problems. Patients reported that having an informed advocate accompany them on hospital visits helped them remember and interpret information provided by the healthcare providers. Peer support is also very helpful at the time of diagnosis, when many patients feel entirely alone and unsupported, and Crohn's and Colitis Canada has taken the initiative in piloting an online peer support program to help alleviate some of this isolation and anxiety.

Summit participants encouraged Crohn's and Colitis Canada to continue with their outstanding work in providing essential information for IBD patients, their families, and caregivers, as well as their efforts to increase awareness. Patients were enthusiastic about the launch of the GoHere Washroom Access Initiative, which will use a GoHere decal, mobile app, and access card to persuade businesses to open their bathroom facilities to people living with IBD and other incontinence issues. The initiative will pave the way for a dialogue on the development of legislation on washroom access, such as what already exists in several US states and in New Zealand. Sustained advocacy is important to educate politicians and governments on the growing disease burden and the challenges faced by patients and their caregivers. Increased awareness will result in better quality of life for people living with IBD.

In hospitals and other healthcare facilities, patients called for designated IBD treatment areas similar to specialized areas for the treatment of cancer. At a minimum, patients need access to additional bathroom facilities and specialized IBD staff who understand the disease. Some larger centres already have dedicated IBD clinics and inpatient wards, a practice that patients suggested should become more widespread. Adequate documentation of patient health and medical needs in the form of an electronic patient chart, mobile health application (mHealth app), or paper summary would also be helpful. Another suggestion was for letter templates verifying special needs, such as bathroom access, for signature by healthcare providers. Summit participants recognized that expanding the number of IBD clinics would present a challenge for strained healthcare resources and would require advocacy efforts aimed at policy-makers and hospital administrators to prioritize IBD.

## 4. Unique Challenges of Children with IBD: Navigating Life Transitions

Despite the high standard of care provided to Canadian children with IBD, patients felt that there is room for improvement. Only a few urban centres currently have dedicated pediatric IBD clinics and programs with ready access to experienced and specialized health professionals, such as physicians, nurses, dieticians, social workers, and psychologists. Multidisciplinary and specialized IBD clinics were felt to be of the highest priority in the care of the rapidly growing population of children with IBD. In rural and northern areas of the country there is often a scarcity of health practitioners experienced in childhood-onset IBD, leading to delays in diagnosis and timely access to effective therapy. Often patients travel long distances to urban centres to get the necessary care, increasing the stress on the family, as well as the financial burden.

Parents' views differed on whether the school system adequately met the needs of children with IBD. Active parental involvement in educating staff about the special requirements of children with IBD, including the social consequences of needing immediate bathroom access, was identified as key to ensuring understanding and appropriate care for children with IBD. As children get older and more independent, however, it becomes necessary for them to take more responsibility. They must ensure that teachers are aware of their condition and special needs before it becomes necessary to request more time for exams or a leave of absence.

Coping with life transitions can be challenging, even for healthy children. This is especially true as children transition from pediatric to adult care. Often this coincides with the start of postsecondary education, and leaving home. The child must become their own advocate at a time that is filled with competing priorities. Sometimes an adolescent who was in remission when they left home requires care in a new city but has no idea how to find a gastroenterologist. The need for help extends well beyond the first year of transition and may continue up to 25 years of age, and beyond, through a series of changing priorities and life events. One patient recounted the story of her son who, despite having a parent with a long history of CD, faced challenges in getting his symptoms taken seriously. After three years, his persistence finally paid off and he was diagnosed with CD at age 29 years.

*Recommendations*. Specialized and multidisciplinary IBD clinics should be a priority of pediatric health centres across Canada. In addition, structured transition programs should be provided to adolescents transitioning to adult care. An important factor is the availability of a dedicated staff person to provide an anchor at transition time, as well as the presence of trained specialist IBD nurses in the clinic. There should be continued emphasis on research to determine the key elements of a successful transition.

Although Crohn's and Colitis Canada has recently developed a resource guide for teachers and a youth webinar series that focuses on issues impacting students with IBD, it was suggested that even more written information for schools and universities would be helpful. In addition, a program to provide speakers on professional days to raise awareness about the challenges faced by young people living with IBD would be a valuable asset.

## 5. Access to Medications

The variety and effectiveness of medications used to treat IBD have improved dramatically in recent years, particularly with the introduction of biological therapies. While improving overall outcomes, the cost of these new medications is a challenge for many patients. At particular risk are low-income families, those without adequate medication insurance coverage, and young adults transitioning from parental coverage who may find it difficult to get insurance for a preexisting condition. Sometimes patients stop taking necessary medications because they are unable to afford them. In addition, some insurance companies and public drug plans require a patient to fail one drug before approving coverage for a more effective treatment. There is considerable variability among the provinces with respect to drug coverage and access, resulting in inequity across the country. Despite the fact that Canada is perceived as a model for patient input into the drug approval processes, many challenges remain.

*Recommendations*. Some provinces have special reimbursement programs, such as the Ontario Trillium Benefit [6], which helps underinsured people with high prescription drug costs relative to their income. For drugs that are not approved under provincial drug plans, there is also the Exceptional Access Program. Patient navigators, social workers, and other experts can help facilitate and speed the process of applying for such funding programs. Summit participants recommended improved emphasis on this role in supporting IBD patients.

Summit participants commended Crohn's and Colitis Canada for their current advocacy efforts and encouraged increased activity to improve availability and coverage of new treatments approved for IBD. They suggested the development of a “toolkit” to help patients navigate provincial drug plans and the broader healthcare system. Such a resource could also be used to support advocacy efforts with policy-makers and insurance providers. Consistent messaging will better educate funders on the needs of the IBD community and the cost-benefit ratio of new, expensive biologic medications. Canada has a universal healthcare system but a universal pharmacare plan is needed to provide equitable access to necessary medications for all Canadians and may be cost efficient [7].

## 6. Alternative Treatments

Healthcare professionals recognized that IBD patients increasingly wish to play a more active role in their treatments. One Summit participant described doing his own research on exercise, diet, and supplements and designing a treatment plan, without the support of his healthcare team. Frustration sets in when, after achieving and maintaining remission, a skeptical clinician suggested that the initial diagnosis must have been wrong.

Responding to patient requests for alternative or experimental treatments presents a constant challenge for healthcare providers who genuinely want to accommodate patient needs but not at the expense of jeopardizing their recovery or long-term health. Many alternative treatments are unproven and some have even been proven ineffective (e.g., probiotics in CD). Although dietary management through the use of exclusive enteral nutrition (formula diet) has been shown to induce remission in CD [8], there are no other dietary interventions that have been demonstrated by scientific research to be consistently effective, although anecdotal examples of success exist. Other factors to consider include the feasibility of adopting dramatic sustainable diet and lifestyle changes, as well as an awareness that complete remission including mucosal healing is now the primary goal for better long-term outcomes of patients. Helping the patient “feel better” is no longer acceptable to IBD providers as the only goal of treatment. For their part, patients recognize that evidence that guides therapeutics should be based on well-designed scientific research, such as controlled clinical trials, which do not necessarily account for individual variation or unique populations.

*Recommendations*. As healthcare moves towards a personalized approach it is likely that this will extend to IBD management. Individual biological markers and patient characteristics will be used to guide therapy. This may result in the identification of new effective nonpharmaceutical therapies, such as dietary manipulation, lifestyle changes, and modification of environmental risk factors. Patients were encouraged to discuss alternative approaches with their healthcare team before embarking on individual treatment plans.

Summit participants encouraged Crohn's and Colitis Canada to continue to provide the IBD community with reliable information on the role of diet and alternative therapies in the treatment of IBD. They asked for improved access to qualified, experienced dieticians with specialized IBD training, especially in nonurban regions where access is often challenging.

## 7. Informing Research Priorities

For the medical community, the priorities in IBD are primarily early diagnosis and achieving effective and timely clinical and mucosal remission for improved long-term outcomes. For the research community, obtaining a better understanding of the biology of the disease is the key to identifying preventive strategies, new treatments, and an eventual cure. For IBD patients attending the Summit, priorities included maintaining quality of life while coping with the disease and its treatments.

Summit participants expressed a desire for enhanced patient engagement in setting both adult and pediatric research priorities for IBD to support patient-centred research. While applauding the role of Crohn's and Colitis Canada in supporting valuable Canadian-based IBD research programs, Summit participants identified several areas where they felt that additional research and investment would improve patient outcomes.

Although treatment options have vastly improved over the last decade, patients called for continued research to identify new medications, especially for those patients who have failed medical therapy and are facing surgical interventions. Further research on prescribing practices and treatment algorithms was also encouraged, particularly in assessing the value of using biologic therapies earlier in the course of illness. A major impediment to effective disease control is adherence to treatments, and so research was encouraged on how to impact adherence and better educate patients about their disease and the importance of adherence to the current medications. Some participants also felt strongly that more research is needed on dietary and alternative treatments. Participants welcomed the trend towards personalized medicine, as patient responses to environmental factors and therapies are highly variable. Patients encouraged multidisciplinary research to support an integrated approach for people with multiple health conditions to assess contraindications for certain drugs and to learn from experiences in other chronic diseases. Capacity building was identified as being essential to support the research effort. This is especially important for students, junior investigators, and clinician scientists to compensate for recent funding cuts by the Canadian Institutes of Health Research [9].

*Recommendations*. While an understanding of the biology of IBD is essential to finding a cure to these diseases, rigorous scientific research should also focus on improving the lives of people living with IBD as well as identifying the underlying environmental and dietary factors which may contribute to disease pathogenesis and exacerbation. While research is needed on natural and dietary therapies, it should follow the same principles of scientific rigour expected of other researches. There is a need for more health services research on optimal models of care for IBD patients, and treatment algorithms. Research is required on the most effective training/mentoring programs for young IBD nurses and the best way to provide peer support for IBD patients in healthcare facilities. Capacity building of young researchers in IBD should be supported to ensure the highest likelihood future advancement.

## 8. Summary of Recommendations

Participants in this Patient and Healthcare Professional Summit recommended efforts to increase awareness of IBD, its complications, and the barriers faced by patients with IBD in daily life. In addition, the challenges facing patients when interacting with the health system should be identified and addressed. These could include, but are not limited to, bathroom access while hospitalized, structured transition programs for adolescents, specialist dieticians, and access to skilled experts to help navigate the health system. Healthcare providers should be more open to discussing personalized treatment plans for patients with IBD, including dietary and alternative therapies, and greater emphasis should be placed on research investigating their role. While curing IBD should remain a priority in IBD research, funding agencies should also encourage the investigation of clinical, health services, and population health research to discover the cause of the diseases, better therapies, and improved health system structures. Capacity building should remain a priority to ensure the skills of the next generation of IBD researchers and healthcare providers.
